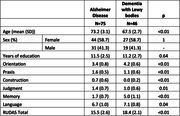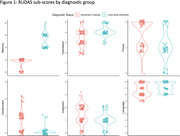# The role of the RUDAS can facilitate in differentiating dementia with Lewy bodies from Alzheimer’s disease in an urban Peruvian population

**DOI:** 10.1002/alz.090494

**Published:** 2025-01-03

**Authors:** Nilton Custodio, Carlos E.E. Araujo Menendez, Belen Custodio, Diego Marco Malaga, Rosa Montesinos, Diego Chambergo‐Michilot, Diego Bustamante‐Paytan, Katherine Aguero, Graciet Verastegui, Giuseppe Tosto

**Affiliations:** ^1^ Research unit, Instituto Peruano de Neurociencias, Lima Peru; ^2^ Escuela Profesional de Medicina Humana, Universidad Privada San Juan Bautista, Lima, LIMA Peru; ^3^ Cognitive Impairment Diagnosis and Dementia Prevention Unit, Peruvian Institute of Neurosciences, Lima, Lima Peru; ^4^ Instituto Peruano de Neurociencias, Lima, Lima Peru; ^5^ University of California, San Diego, La Jolla, CA USA; ^6^ Instituto Peruano de Neurociencias, Lima Peru; ^7^ Unidad de diagnóstico de deterioro cognitivo y prevención de demencia, Instituto Peruano de Neurociencias, Lima, Perú, Lima, Lima Peru; ^8^ Facultad de Ciencias de la Salud, Universidad Científica del Sur, Lima Peru; ^9^ Gertrude H. Sergievsky Center, Taub Institute for Research on the Aging Brain, Departments of Neurology, Psychiatry, and Epidemiology, College of Physicians and Surgeons, Columbia University, New York, NY USA; ^10^ Taub Institute for Research on Alzheimer’s Disease and the Aging Brain, Vagelos College of Physicians and Surgeons, Columbia University, New York, NY USA; ^11^ Department of Neurology, Vagelos College of Physicians and Surgeons, Columbia University, and the New York Presbyterian Hospital, New York, NY USA

## Abstract

**Background:**

Dementia with Lewy bodies (DLB) and Alzheimer’s disease (AD) differ in their cognitive characteristics. Subscales of the Rowland Universal Dementia Assessment Scale (RUDAS) provide an opportunity to test these cognitive domains across the two conditions. Multiple brief cognitive screening tools have been evaluated to differentiate specific types of dementia, but there are no reports regarding RUDAS yet.

**Methods:**

One hundred and twenty‐one [DLB (n = 46) and AD (n = 75)] patients enrolled in the Instituto Peruano de Neurociencias (IPN). AD cases were derived from the GAPP study (NIA grant #alz090494AG069118). Independent group t‐tests and chi‐square tests were used to examine differences in key demographic variables and in RUDAS sub‐scores (Orientation, praxis, construction, judgment, memory, and language) between groups.

**Results:**

On average, AD patients were older and had a lower total RUDAS score. AD and DLB patients performed significantly differently (*p < 0.05*) on all RUDAS cognitive domains (table 1). The RUDAS memory sub‐score was particularly effective in fully differentiating patients with AD from ones with DLB (AD: mean = 1.7, range = 0‐2; DLB: mean = 5, range = 4‐8; Figure 1).

**Conclusion:**

Our results provide preliminary evidence of the utility of RUDAS to differentiate between cases diagnosed with AD and DLB in urban Peruvian populations. Future work will focus on confirming if the RUDAS memory sub‐score can aid in differentiating DLB from AD in larger, community‐dwelling populations in Peru.